# Anastrozole monotherapy further improves near-adult height after the initial combined treatment with leuprorelin and anastrozole in early-maturing girls with compromised growth prediction: results from the second phase of the GAIL study

**DOI:** 10.3389/fendo.2024.1366970

**Published:** 2024-04-02

**Authors:** Dimitrios T. Papadimitriou, Eleni Dermitzaki, Panagiotis Christopoulos, Sarantis Livadas, Ioanna N. Grivea, George Mastorakos

**Affiliations:** ^1^ Department of Pediatrics, Faculty of Medicine, University of Thessaly, Larisa, Greece; ^2^ Aretaieion University Hospital, National and Kapodistrian University of Athens, Athens, Greece; ^3^ Department of Pediatric and Adolescent Endocrinology, Athens Medical Center, Marousi, Greece; ^4^ Hellenic Endocrine Network, Athens, Greece

**Keywords:** aromatase inhibitors, anastrozole, early puberty, precocious puberty, adult height, LHRH analogue, girls, GAIL study

## Abstract

**Background:**

The first phase of the GAIL study (“Girls treated with an Aromatase Inhibitor and Leuprorelin,” ISRCTN11469487) has shown that the combination of anastrozole and leuprorelin for 24 months is safe and effective in improving the predicted adult height (PAH) in girls with early puberty and compromised growth prediction by +1.21 standard deviation score (SDS; +7.51 cm) compared to inhibition of puberty alone, +0.31 SDS (+1.92 cm).

**Objectives and hypotheses:**

In the second phase of the GAIL study, we assessed the adult height (AH)/near-adult height (NAH) at the end of the first phase and, in addition, the efficacy of anastrozole monotherapy thereafter in further improving NAH.

**Methods:**

We measured the AH (age 16.5 years)/NAH [bone age (BA), 15 years] of the 40 girls included, divided into two matched groups: group A (20 girls on anastrozole + leuprorelin) and group B (20 girls on leuprorelin alone). Group A was further randomized into two subgroups: A1 and A2. Group A1 (*n* = 10), after completion of the combined therapy, received anastrozole 1 mg/day as monotherapy until BA 14 years, with a 6-month follow-up. Group A2 (*n* = 10) and group B (*n* = 20), who received only the combined treatment and leuprorelin alone, respectively, were recalled for evaluation of AH/NAH.

**Results:**

AH or NAH exceeded the PAH at the completion of the 2-year initial phase of the GAIL study in all groups, but the results were statistically significant only in group A1: NAH–PAH group A1, +3.85 cm (+0.62 SDS, *p* = 0.01); group A2, +1.6 cm (+0.26 SDS, *p* = 0.26); and group B, +1.7 cm (+0.3 SDS, *p* = 0.08). The gain in group A1 was significantly greater than that in group A2 (*p* = 0.04) and in group B (*p* = 0.03). Anastrozole was determined to be safe even as monotherapy in Group A1.

**Conclusions:**

In early-maturing girls with compromised growth potential, the combined treatment with leuprorelin and anastrozole for 2 years or until the age of 11 years resulted in a total gain in height of +9.7 cm when continuing anastrozole monotherapy until the attainment of NAH, as opposed to +7.4 cm if they do not continue with the anastrozole monotherapy and +3.6 cm when treated with leuprorelin alone. Thus, the combined intervention ends at the shortest distance from the target height if continued with anastrozole monotherapy until BA 14 years.

## 1 Introduction

The GAIL study (“Girls treated with an Aromatase Inhibitor and Leuprorelin,” ISRCTN11469487) was a prospective phase IIa study assessing 40 girls consecutively referred for early puberty (onset, 7.5–9 years) with a predicted adult height (PAH) less than −2 or >1.5 SD lower than their target height (TH) (1). All these girls had initiated central puberty, but very few of them might have had precocious puberty, according to the age limit of 7.5 years, as defined by Greek data to distinguish precocious from early puberty (2). In the first phase of the GAIL study, 20 girls were treated with leuprorelin 11.25 mg depot injection (3) plus anastrozole, and 20 girls were treated with leuprorelin alone for 2 years or until the age of 11 years, as further continuation of pubertal inhibition after the age of 11 years might have resulted in a loss rather than a further gain as far as PAH is concerned, which is in accordance with Carel et al. (4). The two groups did not differ in age, height, body mass index (BMI), bone age advancement (BAA), TH, or distance of PAH from TH. Their bone age (BA) was inappropriately advanced compared to their TH percentile, which was higher than the percentile on which they were growing, as these girls did not follow the pattern of constitutional advancement of growth and puberty (CAGP) (5), which is the major determinant of precocious or early puberty. The first phase of the study clearly showed that the combination of anastrozole and leuprorelin for up to 24 months and until the age of 11 years was safe and effective in ameliorating PAH in girls with early puberty and compromised growth by +1.21 standard deviation score (SDS; +7.51 cm) compared to inhibition of puberty alone, +0.31 SDS (+1.92 cm). Although these results were straightforward, they dealt only with PAH, and the real impact of this strategy on adult height (AH) per se or at least near-adult height (NAH) remains open.

Thus, in the second phase of the GAIL study, we studied whether the gain attained in PAH was indeed preserved after cessation of the combined treatment and translated into a real increase in AH/NAH and whether the continuation of anastrozole monotherapy until BA 14 years resulted in a further improvement in AH, or at least NAH. We also evaluated the safety of anastrozole as monotherapy in these girls.

## 2 Materials and methods

At the end of the combined treatment with leuprorelin and anastrozole, the 20 girls in Group A of the GAIL study (1) were further randomized into two subgroups using their electronic health record numbers only (6). A total of 10 girls forming subgroup A1 continued anastrozole as monotherapy until BA 14 years, while the 10 girls forming subgroup A2 did not receive any therapy. Assignment to either group was presented as our medical decision as this was an open-label trial. The two subgroups did not differ in median age, BMI, TH, and PAH (*p* < 0.05), as shown in **Table 1** (1).

**Table 1:** Characteristics of the girls in subgroup A1 (who continued anastrozole as monotherapy until bone age 14 years) and subgroup A2 (who did not receive any therapy after completion of the first phase of the GAIL study) at the inclusion of the second phase of the GAIL study.

	Age (years)	BMI (SDS)	TH (cm)	PAH (cm)
Group A1 (*n* = 10)	11.0	1.18	160.98	152.36
Group A2 (*n* = 10)	10.7	1.16	161.33	153.92
*p*-value	*0.23*	*0.12*	*0.31*	*0.31*

For age, the values shown are medians.

BMI, body mass index; SDS, standard deviation score; TH, target height; PAH, predicted adult height.

Treatment with anastrozole tablets was at the dose of 1 mg (p.o.) once daily (Arimidex^®^). Patients in subgroup A1 were followed at 6-month intervals. The patients and their parents were advised to report any sign of hyperandrogenism (e.g., acne, hirsutism, or hair loss) and incidents of peculiar feelings or behavior. Medication was electronically prescribed as an off-label treatment, with the costs covered by the patients’ social security at 75%, which ensured treatment compliance.

In the follow-up visits, a complete physical examination with accurate height measurements, pubertal Tanner staging, a BA X-ray, a pelvic ultrasound by a pediatric radiologist, and biochemical testing (at 0800 hours and after an overnight fast) were obtained. Dual-energy X-ray absorptiometry (DEXA) and anterior–posterior/lateral X-rays of the lumbar spine were performed annually. All the methodology and statistical analyses of the GAIL study were followed as previously presented and published in detail (1).

NAH was defined as the height at BA 15 years (7) and adult (final) height as the age or BA at 16.5 years (8).

All procedures were in accordance with the ethical standards and with the approval of the institutional research committees as described in the BMC ISRCTN registry (ISRCTN11469487, https://doi.org/10.1186/ISRCTN11469487), as the second phase of the GAIL study with anastrozole monotherapy was included in the original GAIL study design. Additional scientific and ethical approval (No. 6762) was also obtained from the relevant committee of the University General Hospital of Larisa, Greece, for the second phase of the GAIL study. Informed consent was obtained from the parents of all individual participants.

## 3 Results

The results on the median AH/NAH (in centimeters) and the distance from TH (NAH–TH, in centimeters) at the end of the GAIL study compared to the first phase inclusion and its end are shown in **Table 2**. In early-maturing girls with compromised growth, initial treatment for 2 years or until the age of 11 years with leuprorelin 11.25 mg/12 weeks + anastrozole 1 mg/day resulted in a gain of +9.7 cm in total when treated with anastrozole monotherapy until BA 14 years, which was +2.3 cm more than the gain of +7.4 cm if they did not continue with anastrozole monotherapy and +6.1 cm more than those treated with a luteinizing hormone–releasing hormone analog (LHRHa) alone, who gained only +3.6 cm. The combined therapy continued with anastrozole monotherapy, which ended in the shortest distance of NAH from TH, i.e., −4.7 cm (from −14.48 at inclusion) compared to −5.7 cm (from −13.48 at inclusion) in those with LHRHa + anastrozole alone and −8.7 cm (from −12.82 at inclusion) in girls treated with LHRHa alone. AH or NAH exceeded that of the PAH at the completion of the first phase of the GAIL study in all three groups, but the results were statistically significant only for group A1: NAH–PAH group A1, +3.85 cm (+0.62 SDS, *p* = 0.01); group A2, +1.6 cm (+0.26 SDS, *p* = 0.26); group B, +1.7 cm (+0.3 SDS, *p* = 0.08). The extra gain in height of group A1 was significantly higher than that of group A2 (*p* = 0.04) and of group B (*p* = 0.03). It has to be noted that while there was a significant difference between the A1 and A2 groups at the end of the first phase when their PAH had a greater distance from the NAH, these groups finally had better growth and reached a shorter distance from their TH compared to the A2 group (3.85 *vs*. 1.66 cm, *p* = 0.04), indicating that anastrozole monotherapy could have been even more effective than it appears when comparing only the AH/NAH.

**Table 2:** Results on the median adult height/near-adult height (NAH) and the distance from target height (NAH–TH) at the end of the GAIL study compared to the initial (first phase inclusion and its end).

Group	PAH at initial (first phase inclusion)	PAH (end of the first phase)	NAH (cm)	NAH–PAH ((end of the first phase)	NAH–PAH at initial (first phase inclusion)	TH–NAH (cm)
A1	146.5	152.36	156.21	3.85	9.7	4.7
*p*-value		*0.01*		*0.001*		
A2	148.1	153.92	155.58	1.66	7.4	5.7
*p*-value		*0.26*		*0.006*		
*p*-value (A1 *vs*. A2)	*0.11*	*0.12*		*0.04*	*0.03*	
B	151.08	153.0	154.7	1.7	3.6	8.7
*p*-value		*0.08*		*0.004*		
*p*-value (A1 *vs*. B)				*0.03*	*0.002*	*0.01*
*p*-value (A2 *vs*. B)				*0.47*	*0.020*	*0.02*

Group A1: 10 girls that continued anastrozole as monotherapy until BA 14 years.

Group A2: 10 girls that did not receive any therapy after completion of the first phase of the GAIL study.

Group B: 20 girls on leuprorelin alone at the first phase of the GAIL study.

**Figure 1:**
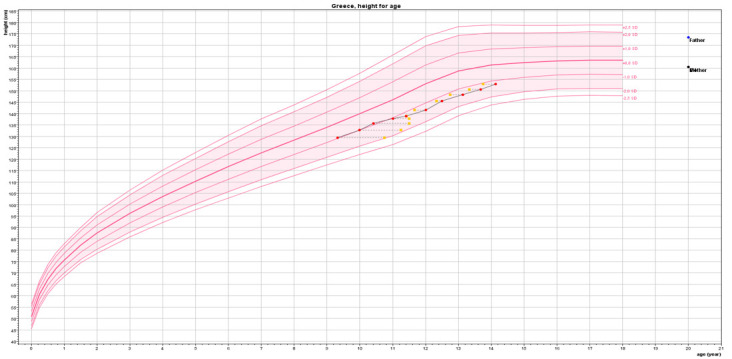
Virtual growth curve of the girls included in the GAIL study based on the median age, height, and bone age of the girls treated with leuprorelin + anastrozole until the age of 11 years and anastrozole monotherapy thereafter until bone age 14 years.

The evolution of the PAH, BAA, BMI, and height velocity (HV) of the subjects in subgroup A1 is presented in **Table 3**. The PAH was significantly higher in the girls in subgroup A1 at 24 months (155.9 cm, *p* = 0.04) and 30 months (156.34 cm, *p* = 0.03) of treatment compared to the PAH of 152.36 cm at the beginning of this second phase of the GAIL study. This was achieved due to the reduction in the advancement rate of BA, practically extending the growth period in combination with the increase in the girls’ height velocity SDS (statistically significant at 12, 18, 24, and 30 months). Anastrozole monotherapy until BA 14 years further improved the AH/NAH by +3.85 cm (+0.62 SDS, *p* = 0.001). The gain in BAA that was achieved with the combined therapy in the initial phase of the GAIL study was preserved until completion of growth, with the BA even lagging behind the chronological age in the first 6 months with anastrozole monotherapy (**Figure 1**). Thus, the greatest effect on BA advancement appears to be that of the combined LHRHa + anastrozole treatment. The HV presented a gradual increase in each 6-month follow-up visit, becoming statistically significant at the third visit at 12 months (**Table 3**). This was due to the initiation of a pubertal growth spurt after the cessation of pubertal inhibition. The BMI SDS remained unchanged (**Table 3**).

**Table 3:** Evolution of the median predicted adult height (PAH), bone age advancement (BAA; delta bone age/chronological age), body mass index [BMI, standard deviation score (SDS)], and height velocity (HV, SDS) of subgroup A1 on anastrozole monotherapy.

	Inclusion	6 months	12 months	18 months	24 months	30 months
PAH (cm)	152.36	154.00	155.17	155.4	155.9	156.34
*p*-value		*0.18*	*0.059*	*0.06*	*0.04*	*0.03*
BAA (years)	0.14	−0.24	−0.21	−0.22	−0.23	−0.29
*p*-value		*0.12*	*0.16*	*0.12*	*0.16*	*0.11*
BMI	1.14	1.03	0.90	0.96	0.91	0.97
*p*-value		*0.35*	*0.20*	*0.29*	*0.23*	*0.31*
HV	−3.44	−3.39	−0.73	−0.42	0.94	2.58
*p*-value		*0.47*	*0.002*	*<0.001*	*<0.001*	*<0.001*

None of the girls presented clinical signs of hyperandrogenism (e.g., acne, hirsutism, or hair loss). The testosterone concentrations are shown in **Table 4**. Testosterone rose slightly above 0.5 ng/ml in three girls, but none developed clinical hyperandrogenism. One girl presented with ovarian stromal hyperplasia, with an ovarian volume slightly above 10 ml in pelvic ultrasound compatible with a polycystic ovary syndrome image, but without any cycle disturbances or any biochemical or clinical signs of hyperandrogenism. Overall, the hematocrit, lipid, and biochemical profiles did not change significantly during treatment. None reported any adverse events, nor were there any signs of emotional instability reported. The DEXA scans showed normal median (range) bone mineral density (BMD) *z*-scores for BA without significant inter-patient changes: −0.4 (−0.5 to 1.0) at inclusion, −0.4 (−0.5 to 1) at 1 year, and −0.3 (−0.4 to 1.1) at 2 years on anastrozole monotherapy. The time to menarche data after discontinuation of leuprorelin were compared between the three subgroups and were found to be practically identical [(median (range), years]: group A1 at 12.3 (11.65–12.8), group A2 at 12.3 (11.2–12.75), and group B at 11.9 (11.3–13).

**Table 4:** Evolution (average ± SD) of the testosterone concentrations in Group A1.

	Inclusion	6 months	12 months	18 months	24 months	30 months
Testosterone (ng/ml)	0.23 ± 0.14	0.3 ± 0.17	0.38 ± 0.20	0.33 ± 0.14	0.37 ± 0.19	0.32 ± 0.04
*p*-value		*0.18*	*0.06*	*0.08*	*0.06*	*0.15*

## 4 Discussion

In early-maturing girls with compromised growth, continuation of anastrozole monotherapy until BA 14 years after an initial combined treatment with leuprorelin and anastrozole for 2 years or until the age of 11 years not only preserved the initial gain in PAH but also further improved it, with a statistically significant further gain of +3.85 cm, corresponding to +2.3 cm more in NAH than in those who completed only the initial combined treatment. Thus, the aromatase inhibitor (AI) anastrozole appears to be an effective and safe treatment even as monotherapy in ameliorating NAH in girls with accelerated BA and a compromised growth prediction. The fact that the initial combined treatment with leuprorelin and anastrozole resulted in a total height gain of +9.7 cm when continuing anastrozole monotherapy until attainment of NAH, compared to +7.4 cm if the combined treatment is stopped and only +3.6 cm when treated with leuprorelin alone, clearly shows that the combined intervention continued with anastrozole monotherapy ends at the shortest distance from TH. These girls reached and probably exceeded (as NAH leaves a margin for an additional 2% until AH is attained) the total pubertal gain of around 27 cm expected in normal Greek girls (9). Thus, the addition of anastrozole to an LHRHa, apart from being safe, is effective in substantially ameliorating the NAH of girls with early puberty and compromised growth potential, making the intervention meaningful.

The GAIL study has several limitations, which have been thoroughly discussed (1), the most significant being the study design, as this is not a randomized double-blind placebo-controlled trial. Another extremely important one is the limited number of patients included, especially in the second phase. However, the study design simulated randomization in its first phase as close as possible to the real-world setting in practicing clinical pediatric endocrinology, and a control group was included. In its second phase with anastrozole monotherapy, randomization was absolute, but based on electronic health records only. To our knowledge, this is the first study to show data on AH or NAH in early-maturing girls with a compromised growth potential who were treated with an initial combination of an LHRHa and an AI for 2 years or until the age of 11 years, and then with an AI alone as monotherapy or with no further therapy until practical completion of growth, i.e., BA 14 years, compared to a control group treated alone with an LHRHa for 2 years or up to the age of 11 years.

The frequency of visits to pediatric endocrinology outpatient clinics for precocious puberty is rapidly increasing (10), particularly for girls (2). The recognition of the pattern of CAGP by general physicians following children and pediatricians apart from specialists in pediatric endocrinology is therefore of fundamental importance (11). This pattern, which is the mirror image of the constitutional delay of growth and puberty (CDGP) (12, 13), is the major determinant of borderline precocious or early puberty in girls (14) and is associated with early onset of adiposity rebound and obesity (11, 15), which can lead to premature adrenarche (16), which is linked to early thelarche and menarche (17). The improvement in socioeconomic conditions that took place in the second half of the 20th century resulted in an earlier onset of puberty in children (18), with a decrease in the age at menarche, leveling off, however, at least in developed countries (9).

Thus, a significant proportion of children presenting with concerns about early pubertal development represent physiological variations that do not require treatment (19). The major concern, as in the girls treated in the GAIL study, is the compromised AH prediction due to advanced skeletal maturation. This is why therapeutic interventions must be timely and individualized, and ideally, gonadotropin-releasing hormone analogs (GnRHa) must be started before the initiation of the growth spurt (20). Most studies agree that inhibition of puberty is useful and effective in progressive precocious puberty (21). However, the subset of precocious, slowly progressive puberty probably corresponds to the pattern of CAGP, which is a normal variation of growth and pubertal maturation and does not require pubertal inhibition (22–24). Similarly, early puberty starting at 7.5–8.5 years with an initial normal height prediction does not require pubertal inhibition (25). Girls with advanced progressive puberty starting between 8 and 9 years of age (26) and advanced-normal puberty with onset between 8.5 and 10 years (27) with an initial height prediction 3–5 cm lower than their TH (practically up to −1 SD) have been found to have no benefit in final height from pubertal inhibition, practically reaching their TH even without treatment, showing that gonadotropin-suppressive therapy in the above groups affects the pace of puberty but not the total pubertal growth or final height. However, in selected girls with rapidly progressive borderline early puberty starting between 7 and 10 years, treatment with GnRHa may be considered (28). If untreated, these girls were found to lose 3.6 cm compared to normal controls, which was exactly the real gain found in group B in the GAIL study, i.e., in the girls treated with leuprorelin alone.

However, a subset of girls—and boys probably so—with constitutional–idiopathic short stature and normal early pubertal development (29, 30) growing with a normal HV at or even below their projected TH curve, initially implying a possible pattern of CDGP, ultimately begin their pubertal maturation either at a particularly low height and/or with a markedly advanced or at least not delayed BA without presenting an early-onset growth spurt. These children, if untreated, reach AHs considerably lower than their THs and at the lower end or below normal for the population. This is exactly the subset of children included in the GAIL study.

Third-generation AIs have been used to increase the PAH in boys (31–34), in girls in the context of McCune–Albright syndrome (35), and, recently, even in girls with congenital adrenal hyperplasia (CAH) (36). The concept of using AIs in girls lies in the fact that peripheral aromatization of mainly the adrenal but also ovarian androgens is the main mechanism of BAA (37), with extragonadal estrogen biosynthesis, particularly in the bone, deploying a “paracrine” or “intracrine” action (38). An increase in AH can be attained in growing adolescents by inhibiting estrogen action, providing a rationale for studies aimed at delaying the maturation of growth plates and increasing AH when the growth potential is compromised (39). The use of selective inhibitors of the aromatase enzyme with AIs (also in combination with pubertal inhibition and growth hormone) represent therapeutic choices that have been studied as strategies to maximize pubertal growth in children with compromised growth (33). Anastrozole appears to be more effective in slowing epiphyseal maturation and in increasing PAH, even if it is less potent than letrozole (40). Furthermore, previous concerns about the skeletal safety of AIs are gradually subsiding (41), and they have been increasingly used for functional (42) or obesity-induced hypogonadism (43, 44) in men, with anastrozole being the drug of choice for the treatment of infertility in men when using AIs (45), as well as for improving fertility by inducing ovulation in women with polycystic ovary syndrome (PCOS) (46).

Back in 2011, it was stated that “the use of aromatase inhibitors to promote growth in girls should be pursued only in the context of a clinical trial” (34). In 2016, the first phase of the GAIL study showed positive results on the PAH (1); now, after a preliminary report of the second phase in 2020 (47), the results on AH/NAH are finally available. The use of off-label medications in children, however, remains a common practice for pediatric providers (48), and according to the American Academy of Pediatrics policy statement on the use of off-label medications in children (49), “Off-label is the use of a drug that is not included in the package insert (FDA-approved labelling) [and] does not imply improper, illegal contraindicated or investigational use. Off-label use does not necessarily require prescribers to obtain informed consent if the decision to use the medication is supported by scientific or even anecdotal evidence and is not investigational in nature. The purpose of off-label use is to benefit the individual patient and practitioners use their professional judgment to determine these uses. Therapeutic decision-making must always rely on the best available evidence and the importance of the benefit for the individual patient.” While AI therapy may be associated with a positive height outcome, clinicians need to be cautious when counseling families about the potential height outcome with and without intervention, as the difference might be completely unpredictable (50). However, it is important that the use of growth-promoting therapies, including AIs, be found psychosocially beneficial in adolescents with idiopathic short stature (51). The results of the second phase of the GAIL study also imply the possible use of AIs in the treatment of short stature in girls, which is an established off-label treatment in the clinical setting for boys (52).

## 5 Conclusion

While most of the early-maturing girls present a normal variation of CAGP, which does not require treatment, there is a subset who, nonetheless, are not taller or—even worse—are shorter than their projected TH curve with a particularly advanced BA and a severely compromised growth prediction. In these girls, with treatment for 2 years or until the age of 11 years with leuprorelin 11.25 mg/12 weeks combined with anastrozole 1 mg/day (p.o.), the gain in NAH is +9.7 cm in total if treatment is continued with anastrozole monotherapy until BA 14 years, ending in the shortest distance and within the normal range of TH −4.7 cm, compared to +7.4 cm if they do not continue with anastrozole monotherapy (−5.7 cm from TH) and only +3.6 cm (−8.7 cm from TH) when treated with leuprorelin alone. The above findings indicate that while there is some gain with classical pubertal inhibition using the standard approach with LHRHa, an initial combination therapy until 11 years of age, with the AI anastrozole continued as monotherapy until BA 14 years, ends in the shortest distance and within the normal range from TH. This approach is not only safe but also appears particularly effective in substantially ameliorating AH/NAH, making the decision to intervene in the pubertal maturation of these girls incisively meaningful.

## Author’s note

This work is part of the PhD Thesis of E.D.

## Data availability statement

The raw data supporting the conclusions of this article will be made available by the authors, without undue reservation.

## Ethics statement

The studies involving humans were approved by the Athens Medical Center Ethics Committee and General University Hospital of Larisa Scientific Committee. The studies were conducted in accordance with local legislation and institutional requirements. Written informed consent for participation in this study was provided by the participants’ legal guardians/next of kin.

## Author contributions

DP: Conceptualization, Data curation, Formal analysis, Funding acquisition, Investigation, Methodology, Project administration, Resources, Software, Supervision, Validation, Visualization, Writing – original draft, Writing – review & editing. ED: Data curation, Investigation, Methodology, Resources, Writing – original draft, Writing – review & editing. PC: Investigation, Methodology, Resources, Writing – review & editing, Writing – original draft. SL: Formal analysis, Writing – review & editing, Writing – original draft. IG: Supervision, Validation, Visualization, Writing – review & editing, Writing – original draft. GM: Conceptualization, Funding acquisition, Methodology, Project administration, Supervision, Validation, Writing – review & editing, Writing – original draft.
